# Tyrosine Kinase Syk Non-Enzymatic Inhibitors and Potential Anti-Allergic Drug-Like Compounds Discovered by Virtual and *In Vitro* Screening

**DOI:** 10.1371/journal.pone.0021117

**Published:** 2011-06-20

**Authors:** Bruno O. Villoutreix, Guillaume Laconde, David Lagorce, Pierre Martineau, Maria A. Miteva, Piona Dariavach

**Affiliations:** 1 INSERM, U973, (MTi Unit), Université Paris Diderot, Paris, France; 2 IRCM, Institut de Recherche en Cancérologie de Montpellier, Montpellier, France; 3 INSERM, U896, Montpellier, France; 4 Université Montpellier1, Montpellier, France; 5 Université Montpellier2, Montpellier, France; 6 CRLC Val d'Aurelle Paul Lamarque, Montpellier, France; Kyushu Institute of Technology, Japan

## Abstract

In the past decade, the spleen tyrosine kinase (Syk) has shown a high potential for the discovery of new treatments for inflammatory and autoimmune disorders. Pharmacological inhibitors of Syk catalytic site bearing therapeutic potential have been developed, with however limited specificity towards Syk. To address this topic, we opted for the design of drug-like compounds that could impede the interaction of Syk with its cellular partners while maintaining an active kinase protein. To achieve this challenging task, we used the powerful potential of intracellular antibodies for the modulation of cellular functions *in vivo,* combined to structure-based *in silico* screening. In our previous studies, we reported the anti-allergic properties of the intracellular antibody G4G11. With the aim of finding functional mimics of G4G11, we developed an Antibody Displacement Assay and we isolated the drug-like compound **C-13,** with promising *in vivo* anti-allergic activity. The likely binding cavity of this compound is located at the close vicinity of G4G11 epitope, far away from the catalytic site of Syk. Here we report the virtual screen of a collection of 500,000 molecules against this new cavity, which led to the isolation of 1000 compounds subsequently evaluated for their *in vitro* inhibitory effects using the Antibody Displacement Assay. Eighty five compounds were selected and evaluated for their ability to inhibit the liberation of allergic mediators from mast cells. Among them, 10 compounds inhibited degranulation with IC_50_ values ≤10 µM. The most bioactive compounds combine biological activity, significant inhibition of antibody binding and strong affinity for Syk. Moreover, these molecules show a good potential for oral bioavailability and are not kinase catalytic site inhibitors. These bioactive compounds could be used as starting points for the development of new classes of non-enzymatic inhibitors of Syk and for drug discovery endeavour in the field of inflammation related disorders.

## Introduction

Development of novel, safe and effective drugs for the treatment of allergic and autoimmune disorders has been one of the important research goals of pharmaceutical companies in the past decade. Protein therapies such as anti-IgE monoclonal antibody omalizumab (Xolair) for treating allergic airway constriction [1] and TNFα inhibitors in the field of rheumatoic arthritis and chronic inflammatory conditions [2] have shown their high effectiveness, but they can induce side-effects and are expensive therapies.

**Figure 1:**
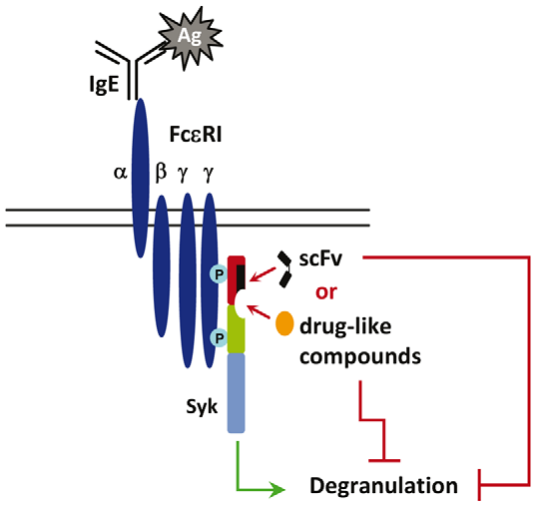
Schematic diagram of mast cell activation. The newly identified cavity of Syk is located at the close vicinity of the binding site of scFv G4G11. The binding of either G4G11 or drug-like compounds to this area inhibit FcεRI-mediated mast cell degranulation.

Targeting proteins that play a key role in signaling pathways, such as adhesion molecules or kinases has been another avenue to address these complex pathologies. Among these targets, the tyrosine kinase Syk has shown a high potential for the discovery of new treatments for inflammatory and autoimmune disorders [3]. Syk is a cytoplasmic protein kinase that is a key mediator of immunoreceptor signaling in B cells, mast cells, macrophages and neutrophils. Syk is activated at the early stages following the stimulation of antigen or Fc receptors at the surface of immune cells, and interacts, via its SH2 domains with a number of substrates that form macromolecular signaling complexes at the plasma membrane, and activates signaling pathways that lead eventually to the inflammatory process (Fig. 1).

Because immunoreceptors including Fc receptors and B cell receptors are important for both allergic and antibody mediated autoimmune diseases, interfering with Syk has been a therapeutic strategy for many pharmaceutical companies. Pharmacological inhibitors of Syk kinase activity bearing therapeutic potential have been developed [3], [4]. One of these compounds, referred to as R112, developed by Rigel, has entered clinical trials and showed remarkable amelioration of allergic rhinitis acute symptoms [5]. An R112-related inhibitor, R406, as well as its orally bioavailable prodrug, fostamatinib (R788, Rigel) are developed for the potential treatment of RA. However, such ATP-competitive kinase inhibitors have limited specificity towards Syk and R406 was shown to inhibit several other kinase and non-kinase targets at concentrations comparable to those inhibiting Syk [6]. On the other hand, because Syk is widely distributed in different cell types, inhibiting its catalytic activity bears the risk of unwanted consequences on various physiological functions such as cell differentiation, adhesion and proliferation [7]. To address this topic, we opted for the inhibition of the interactions of Syk with its cellular partners while maintaining an active kinase protein. For this purpose, we used the powerful potential of intracellular antibodies for the modulation of cellular functions *in vivo* [8].

**Figure 2:**
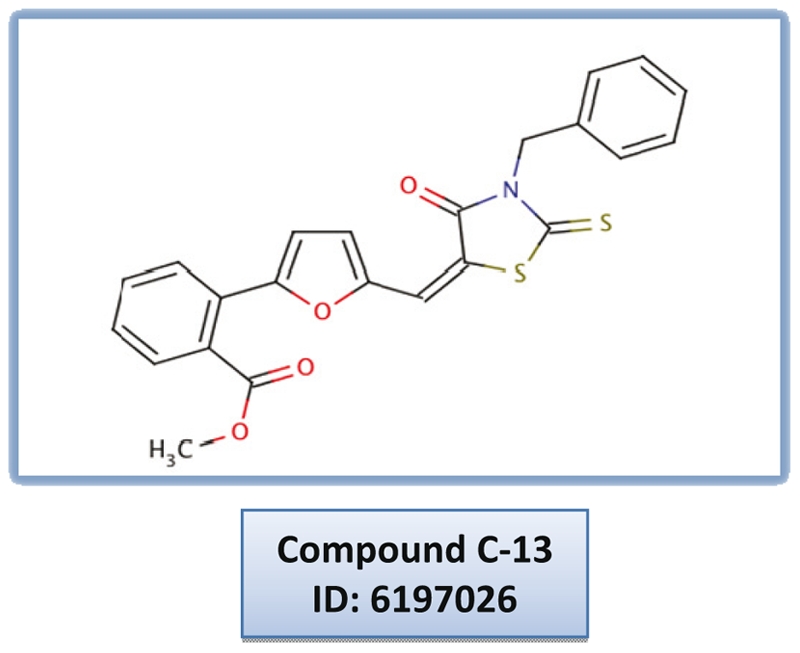
Structure of compound C-13.

We have reported in a previous work [9] the characterization of the anti-Syk intracellular scFv G4G11 that binds to a linear epitope located in the N-terminal SH2 domain of Syk [10]. Our studies showed that intracellular expression of G4G11 does not affect the catalytic activity of the kinase, yet inhibits FcεRI-induced release of allergic mediators in a mast cell line [9] (Fig. 1). With the aim of finding functional mimics of G4G11 that act as potential inhibitors of the allergic response, we developed an approach based on an Antibody Displacement Assay for high-throughput screening of compound collections. Using this approach, we originally screened a diverse library containing 3000 molecules from which we isolated the drug-like compound **C-13** (Fig. 2), a small molecule that inhibits FcεRI-induced mast cell degranulation *in vitro* and anaphylactic shock *in vivo* when administered orally to mice [10]. Structural analysis and site directed mutagenesis allowed us to identify the likely binding cavity of this compound, located at the close vicinity of the scFv G4G11 epitope, at the interface between the two SH2 domains and the interdomain A of Syk (Fig. 1). The screened pocket is distant from the catalytic site, as seen in the low-resolution 3D structure of Syk determined by single particle electron microscopy [11]. Accordingly, our functional studies showed that **C-13** has no effects on the enzymatic activity of Syk, but inhibits the phosphorylation of Syk substrates that form macromolecular signaling complexes at the plasma membrane that are important for the activation of mast cells. We concluded that **C-13** impedes protein-protein interactions of Syk with some of its partners [10].

Although identification of protein-protein interaction inhibitors is a difficult task [12], [13], we believe that it could be a very valuable strategy in the case of Syk. Indeed, several recent success stories indicate that it is possible to find small molecular probes modulating macromolecular complex formation [14], [15]. Thus, in an attempt to identify new non-enzymatic inhibitors of Syk and enlarge our battery of anti-allergic compounds, we performed structure-based virtual screening experiments of a compound collection containing 310,000 molecules after filtering, and targeted the **C-13** cavity in Syk that seems to modulate protein-protein interactions. The top 1000 compounds were evaluated for their *in vitro* inhibitory effects using the Antibody Displacement Assay, and the selected compounds were further characterized *in vivo* in a degranulation assay in mast cells. We identified bioactive compounds different from **C-13** with anti-allergic properties that could be used as starting points for the development of non-enzymatic inhibitors of Syk, that will help to understand some of complex molecular mechanisms involving Syk and for drug discovery endeavour in the field of inflammation related disorders.

## Results and Discussion

### Structure-based virtual screening against a newly identified pocket of Syk

**Figure 3:**
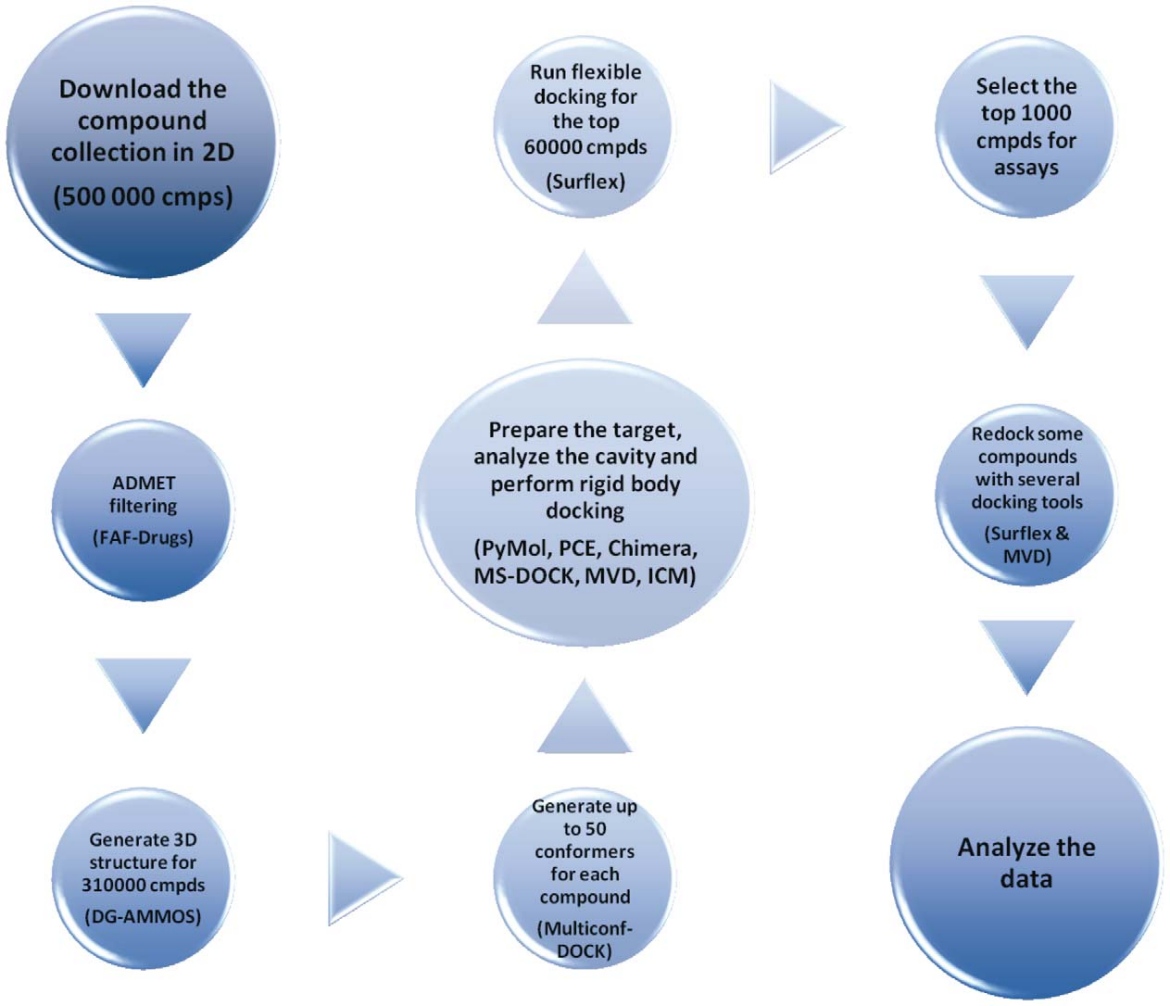
*In silico* screening protocol. Overview of the different steps and computer packages used for the identification of new Syk inhibitors (see text for details).

In a previous work, we reported that scFv G4G11 binds to a region of Syk that is important for the binding of protein partners essential for the degranulation pathway in mast cells [9]. We wished to find drug-like compounds that act as functional mimics of G4G11 and identified, through random experimental screening, the molecule **C-13** (Fig. 2), a small compound able to interfere *in vitro* with the interaction of G4G11 with Syk (see below). We found a binding pocket, with a volume of about 755 A^3^ compatible with the binding of a small drug-like compound [16], [17], far away from the catalytic site and located next to the epitope of G4G11, at the interface between the two SH2 domains and the interdomain A [10]. With this data in hand, we conducted a structure-based virtual screening study to identify new classes of compounds that could bind to this newly identified pocket. We used a hierarchical virtual screening approach [18] that combined the rigid-body docking engine MS-DOCK [19] and Surflex (version 2.514) (incremental construction) [20] (Fig. 3). The ChemBridge compound collection was used after soft ADMET (absorption, distribution, metabolism, excretion and toxicity) filtering and soft assessment for the presence of assay interference, frequent hitter and reactive compounds with the new version of our tool FAF-Drugs [21]. The filtering step removed about 190,000 molecules and the final collection contained 310,000 compounds. The molecules were generated in 3D with DG-AMMOS [22] and Multiconf-DOCK [19]. Rigid body docking was carried out with MS-DOCK [19] in order to select only molecules that would fit in the binding pocket (i.e., geometric filtering step with only at this stage evaluation of shape complementarity). This process was followed by flexible docking with Surflex of the top 60,000 molecules resulting from rigid-body docking. Several groups have reported [18], [23] that such multi-step protocols can speed-up computations while generally giving better results than full flexible docking of the entire collection. This observation applies to several types of binding pockets but not all as, for example, a flat pocket would obviously not benefit from geometric filtering. However, here, in the case of Syk, the pocket was judged appropriate since it forms a relatively pronounced binding groove. The top 2000 molecules after Surflex docking and scoring were analyzed interactively and 1000 were purchased and subjected to *in vitro* and biological assays (Fig. 3).

### Experimental screening of the compound collection

To determine the ability of the compounds to affect the binding of scFv G4G11 to Syk *in vitro*, we tested them individually in an ELISA-based Antibody Displacement Assay (see Materials and Methods). For this purpose, all 1000 compounds were reconstituted in DMSO, and they were tested at a final concentration of 10 µM in PBS. Compound **C-13** was reconstituted in DMF and was used in parallel as a control. The capacity of each compound to inhibit the binding of G4G11 to Syk was evaluated in comparison to the binding of G4G11 to Syk in PBS (final concentration of 100 nM) containing the appropriate concentrations of DMSO or DMF.

This first *in vitro* screen led to the isolation of the 85 most active compounds selected on the basis of their inhibition efficacies ranging from 11% to 86.5% (Table S1). Compound **C-13** used in parallel displayed 81% inhibition. Direct binding to Syk of a selection of these 85 compounds was investigated using fluorescence spectroscopy. The affinity constants ranged from 0.8-21.8 µM and suggested that even compounds with low inhibition efficacies in the Antibody Displacement Assay exhibit good affinities for Syk (Table 1 and Table S1).

**Table 1:** Five most bioactive compounds from cluster 2.

ID ChemBridge	IC50 Cell Degranulation (µM)	Kd (µM)	Solubility (µM)	Lipinski Violations
**6197026 (C-13)**	2	4	ND	1 (log P)
**6282824**	5	5.6	4.5	0
**7495334**	10	1.6	35.2	0
**7501888**	4	8.2	6	2 (log P, MW)
**7752193**	10	8	0.3	0
**7783660**	10	4.8	12.9	1 (MW)

Compound **C-13** is included as a reference.

**Figure 4:**
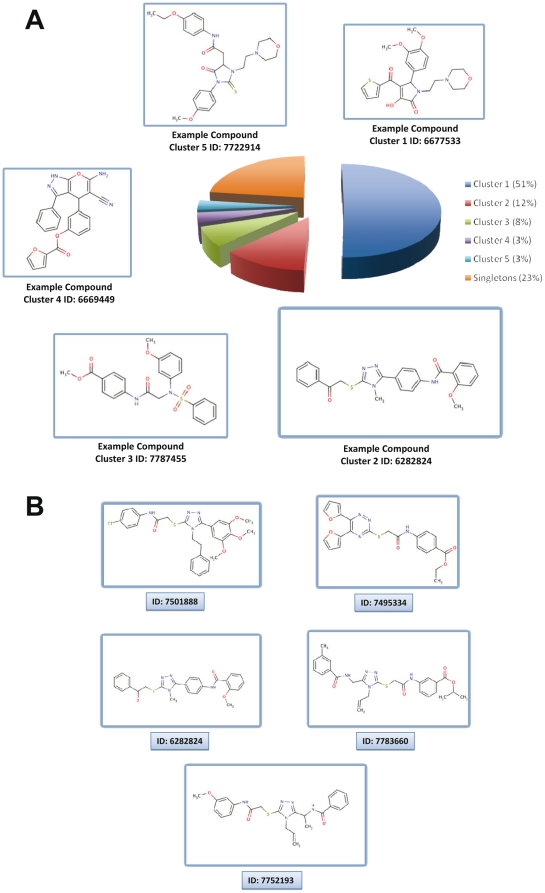
Analysis of the hit compounds inhibiting Syk. (**A**) Pie chart illustration of the 85 selected molecules after similarity search and clustering. (**B**) Structures of the most bioactive molecules from cluster 2.

The 85 compounds were clustered into 5 families and 20 singletons, all different from **C-13**. The two most populated clusters are clusters 1 and 2, which represent 63 % of these 85 molecules (Fig. 4A). Investigation of these two clusters shows the systematic presence in cluster 1 of pyrrol-2-one scaffold while cluster 2 is more diverse and contains compounds with 1,2,4-triazole, quinazolinone, 1,2,4-triazine or dihydrophtalazin-1-one substructure (Fig. 4B). All together, these data suggest that the small molecules were likely to bind in the predicted binding pocket and were either directly (partially protruding onto the epitope area) or indirectly (conformational changes in the epitope area) perturbing the binding of the antibody (see below).

### Biological effects on mast cell degranulation

The 85 compounds were then tested in a cellular functional assay in order to evaluate their ability to inhibit the liberation of allergic mediators from mast cells. The test is based on the measure of the liberation by exocytosis of the enzyme β-hexosaminidase following the stimulation of the FcεRI, the high affinity receptor for IgE at the surface of mast cells. To this aim, RBL-2H3 mast cells were incubated with each compound for one hour at a final concentration of 20 µM, and β-hexosaminidase release following FcεRI stimulation was measured. Among the 85 molecules, 10 compounds inhibited degranulation with IC_50_ values ≤10 µM and interestingly 5 of them belong to cluster 2 (Table 1) and none to cluster 1.

**Figure 5:**
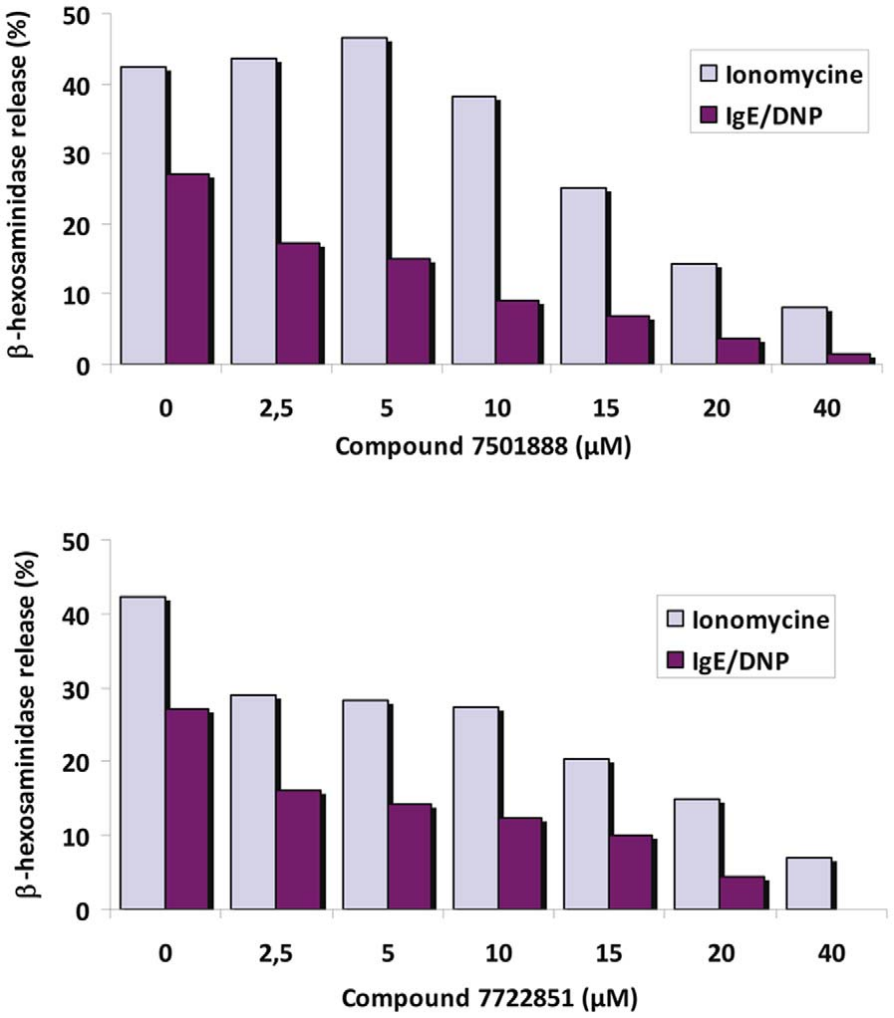
Compounds 7501888 and 7722851 impair FcεRI-induced mast cell degranulation. β-hexosaminidase release from mast cells was evaluated in comparison to ionomycin-induced degranulation as a control of the machinery.

**Figure 6:**
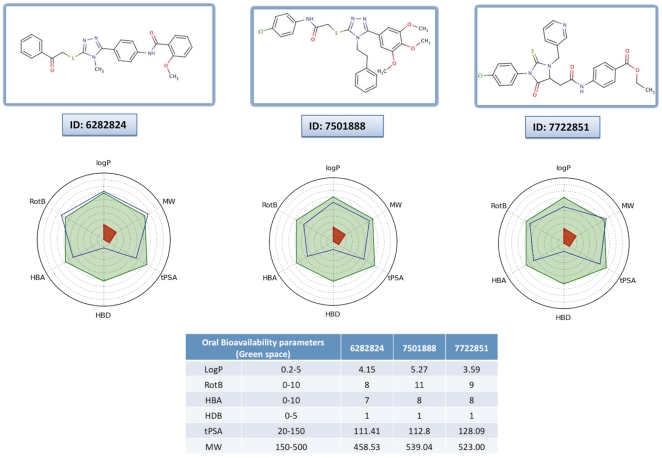
Physico-chemical profile of compounds 6282824, 7501888, 7722851. A radar plot representing the computed oral bioavailability profile (compound blue line should fall within the optimal green area, white and red ones being extreme zones generally indicating low oral bioavailability). This analysis was carried out on all molecules but we present the data only for three compounds. The computations involved: logP, molecular weight (MW), topological polar surface area (tPSA), rotatable bond (RotB), H-bonds acceptors and donors (HBA, HBD).

To address the possible toxic effects of these compounds on degranulation, we performed a dose-dependent experiment in which FcεRI-dependent degranulation of mast cells was performed in parallel with ionomycin-induced degranulation as a control of the degranulation machinery. We illustrate the results of this experiment with two compounds which displayed the best efficacies with IC_50_ evaluated at 4 µM for both: molecule **7501888** which belongs to cluster 2 and molecule **7722851** which belongs to cluster 5. As shown in Fig. 5, while a specific inhibition of the liberation of allergic mediators is observed, the treatment of the cells with ionomycin showed that concentrations up to 10 µM of the compounds had no major toxic effect on mast cells responses. The ADMET profile of our 10 bioactive compounds was analyzed further. This is for instance exemplified taking 3 interesting molecules (compounds **6282824** and **7501888** belong to cluster 2 and compound **7722851** to cluster 5) that combine biological activity (IC_50_ = 4−5 µM), significant inhibition of antibody binding (40–67%) and strong affinity for Syk (Kd = 5−21 µM) as examples. These molecules have overall a good potential for oral bioavailability as seen in Fig. 6. Further, queries into the PubChem database [24] and MDL Drug Data Report (MDDR) (MDL Drug Data Report (MDDR), version 2010; http://accelrys.com/) for these 10 active compounds suggest that these molecules are not toxic while they do not seem to have direct kinase inhibitory activities. In addition, the solubility was measured experimentally and the Lipinski “rule of five” descriptors [25] were computed to probe further the molecules from cluster 2 (Table 1). Taken together, our data suggest that these molecules possess reasonable structures to probe this biological system and for future chemical optimization.

### Structural analysis of selected compounds

**Figure 7:**
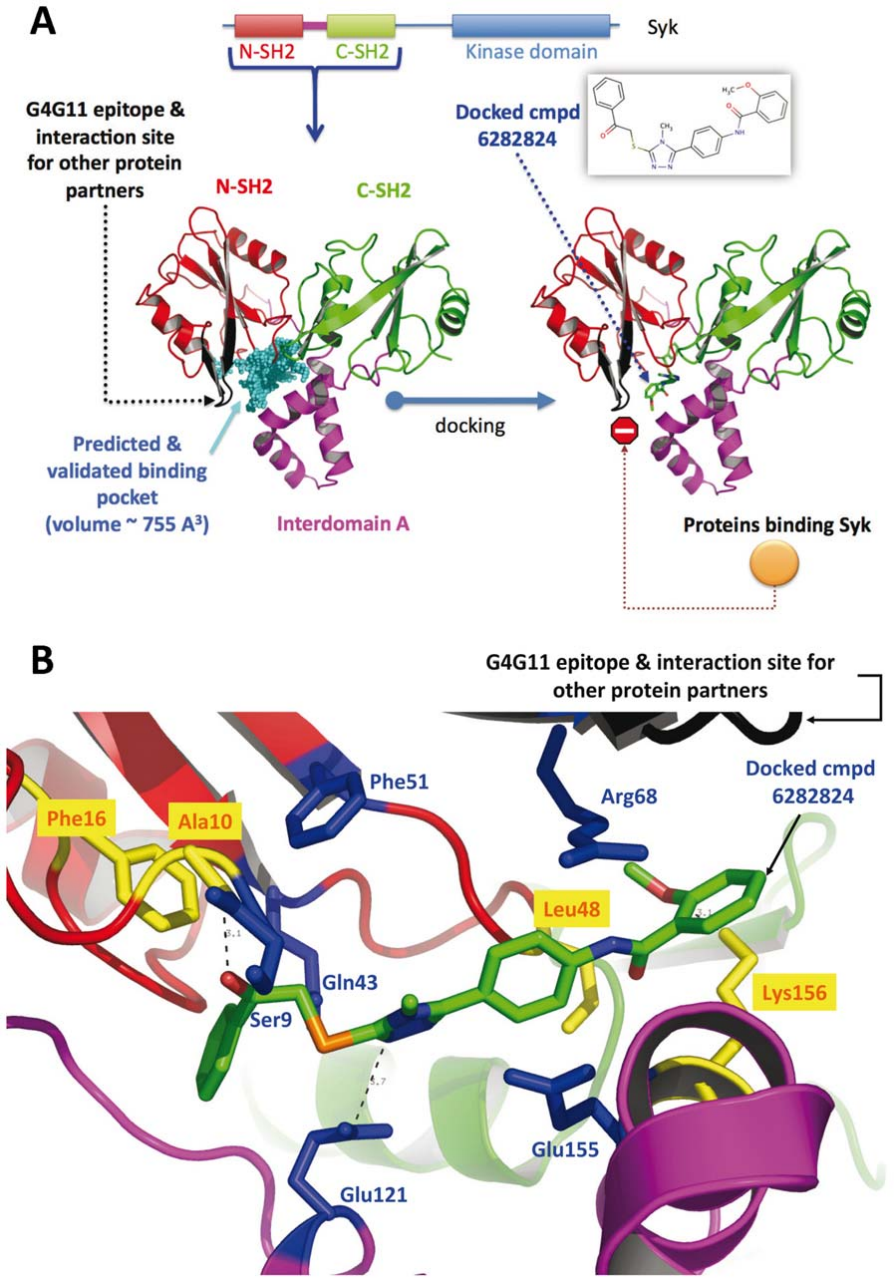
Predicted structure of the complex between compound 6282824 and Syk. (**A**) The small molecule was docked into a binding pocket previously identified by our groups and located far away from the catalytic site. We suggest that the mechanism of action for the small molecule involves either or both, small local structural changes in nearby loops thereby impeding appropriate contacts between Syk and some protein partners or that the small molecule directly clashes into upcoming proteins that interact with Syk. (**B**) Structural analysis of a possible binding pose. A zoom in the binding pocket and rotations were carried out as compared to panel A in order to facilitate the reading of the figure. Side chains mutated in our previous study are shown in dark blue and additional amino acids expected to be important for binding are shown in yellow. The residues found important in our previous mutagenesis study [10] involves Glu 121, Glu 155, and Arg 68. Two hydrogen bonds between the compound and Lys 156 and Ala 10 (backbone N atom) are predicted with a likely electrostatic interaction between Glu 121 and the triazole ring. Hydrophobic, aromatic and pi stacking interactions are also predicted, the main side chains involved are Phe 16, Leu 48, and the hydrophic moiety of the Arg 68 and Glu 155 side chains.

In order to propose a possible mechanism of action for our molecules, we re-docked our best molecules with Surflex (version 2.514) and Molegro Virtual Docker (version 4.3) [26] into the Syk binding pocket. We illustrate the results of the docking experiments with molecule **6282824** but we note that for most compounds, one part of the molecule always seems to be protruding next to the epitope of scFv G4G11 suggesting that they could inhibit the binding of the scFv by steric hindrance (see for example the results for compound **6282824** in Fig. 7). Our previous studies suggested that the area of Syk interacting with the antibody G4G11 is also involved in the interaction of Syk with several, yet not fully elucidated, of its protein partners implicated in the degranulation pathway. The present data suggest that the small molecules fit into the Syk binding pocket, protrude at the molecular surface and could indeed fully or partially inhibit protein-protein interactions and could thus reduce downstream degranulation.

### Significance

Identification of protein-protein interaction inhibitors is a new and very active trend in pharmacology. We here combined *in silico* screening with an *in vitro* screening approach that is based on an antibody displacement assay, to find novel classes of Syk inhibitors that do not bind to its catalytic kinase site but most likely to a cavity located in a region essential for protein-protein interactions. After a series of bioassays, we could identify several drug-like compounds that showed non-enzymatic inhibition of Syk and that could be used as a starting point for medicinal chemistry optimization experiments. These molecules would have valuable applications in the field of inflammation related disorders.

## Materials and Methods

### Virtual screening

We used an efficient multistep virtual screening procedure that is both time- and cost-effective [18], [23]. We selected the 500,000-molecule ChemBridge Corp. compound collection for our study. Soft Absorption, distribution, metabolism, and excretion/tox filtering and assay interference compound analysis was performed with the new version of FAF-Drugs [21]. The remaining 310,000 molecules were docked into a Syk pocket that we have recently discovered and characterized [10]. The binding pocket for docking was defined using several *in silico* procedures including the Surflex protomol utility and by the ICM pocket finder utility (*Molsoft LLC, La Jolla, CA, USA*) and validated by site directed mutagenesis as described by Mazuc et al. [10]. The X-ray structure of Syk SH2 domains, PDB code 1A81, was used [27]. The ICM regularization procedure was applied in order to optimize of hydrogen bond interaction and refine the protein 3D structure. This procedure resulted in two important changes in the structure: reorientation of the side chain of N11, and rebuilding of the K124 side chain, both residues being located in the predicted binding zone. Numerous other small adjustments were also noticed in the structure as expected from the regularization procedure. The protonation state of the histine residues during docking was kept as defined by ICM (nonionized). Additional investigations of the protonation state of titratable groups were performed through electrostatic computations with our PCE server [18], [24] and charges assigned accordingly within Chimera [28]. The electrostatic computations were carried out with two dielectric constants: 4 and 11 for the solute, and 80 for the solvent. Both runs showed normal titration behavior for all titratable groups of the binding zone.

The 3D structures of the small compounds were generated with DG-AMMOS [22] employing a distance geometry method and up to 50 conformers were generated for each ligand with Multiconf-DOCK [19] applying an energy window of 25 kcal.mol^−1^ and a diversity threshold of 0.8 Å RMSD. These values represent an appropriate balance between speed for the subsequent rigid-body docking, and the accuracy in terms of finding bioactive conformations among a conformational ensemble [19], [29], [30]. MS-DOCK is a protocol for multiple conformation rigid body docking that uses the docking engine DOCK [31] in which the score evaluates only the steric complementarity between the ligand and the protein using a contact scoring function that counts the number of receptor-ligand contacts within a 4.5 Å distance from any ligand atom. The allowed bump overlap was chosen to be 0.50 (by default 60) permitting some minor clashes between the atoms of the protein binding pocket and the ligands. It was previously shown that 0.5 is the best bump overlap value in case of a closed and well-formed pocket (see for details [19]), as it is the case here as the targeted zone forms a relatively narrow groove. MS-DOCK is able to successfully reduce the initial chemical library by 2- or 3-fold depending on the binding site shape and volume [19]. Here, because the binding site is a well formed, we suggest that our shape complementarity filter can be used efficiently. After analysis of the pocket and from our previous benchmarking of our MS-DOCK tool, we decided to keep only the top 60,000 compounds scored by MS-DOCK among our compound collection of 310,000 molecules [31].

The flexible docking was then performed on the 60,000 molecules using Surflex v.2.514 with the multistart parameter on (5 starting positions). The docking step was performed using the protomol based on the residue list validated by mutagenesis [10] with an extension of 2 Å (the parameter proto_bloat was set to 2, the default value is 0). For the postprocessing, the following parameters were used: the “polar” term was set to 2 because several charged/polar residues are present in the binding pocket (the default value is 1). We were permissive for penetration violations because the pocket is relatively closed, thus the “crash” value was set to −3.0 (the default value is −1). The top 2,144 docked ligands with a Surflex score superior to 7.0 (i.e., a very good score in Surflex) were analyzed using PyMOL (DeLano, San Carlos, CA) and 1000 molecules were selected after visual inspection and purchased. Keeping in mind that the most active compounds found experimentally have a Surflex score between 9.5 and 7.9, we believe that the applied threshold of 7.0 used in the present study was appropriate for this cavity.

Some selected compounds were re-docked with Surflex v.2.514 turning on several optimization parameters to enhance the search and refine poses [20] and with Molegro Virtual Docker [26]. To re-dock the best active compounds, the following changes were applied to Surflex: the “pgeom” option was used with the clustering RMSD between the poses set to 0.9 Å instead of the default value of 0.5 Å, and 30 poses saved for the structural analysis. Regarding re-dock with Molegro, ligand and protein atoms were prepared within the Molegro package. The detect cavity procedure was applied employing a grid method searching for accessible volumes defined as zones where a sphere probe does not clash with the protein atoms. The cavities found are then ranked according to their volume. The top cavity ranked by Molegro matches with the region probed by site directed mutagenesis. We constrained the search to this cavity by adding a penalty to poses that were found well outside the binding regions. The grid-based scoring function used in this re-docking involves: interaction energy terms between the protein and the ligand (hydrogen bonds, van der Waals interactions, and electrostatic interactions modeled as a Coulomb potential with a distance-dependent dielectric constant), ligand internal energy and, as mentioned above, a penalty if the ligand pose was found to be too far away from the binding cavity (in our case a sphere with a radius of 15 Å around the center of the detected cavity). Ten poses were generated with the RMSD between the poses >1Å. In order to optimize the ligand poses for a more precise structural analysis, we employed several options not activated by default, namely the ligand internal energy and the penalty if the ligand is placed outside of the required zone, and energy minimization of the ligand poses.

The Ligand-info package [32] was used for clustering the 85 compounds that were found to be interesting experimentally. The clustering approach uses two-dimensional structure similarity search. The compounds are represented by modified hashed-fingerprints and the algorithm computes for each molecule a vector of two-dimensional indices as well as their occurrence counts. A Tanimoto similarity index is then used to assess the similarity between all pair of compounds and takes values between 0 and 1, where 0 means that there are no identical indices in either molecule and 1 means that both molecules are composed of identical sets of indices.

### Selection of Drug-Like Compounds (ADMET with FAF-Drugs2)

We decided to use our ADMET filtering package that combines physicochemical filtering and removal of some reactive groups and some compounds that could interfere with assays. Property cutoff values used to select drug-like species follow the ones reported by Irwin and Shoichet [33]: 60< MW <600, 0< HBDonnors <6, 0< HBAcceptors <11, 0< Rotatable Bonds <12, 0 < Rigids Bonds <50, 0< Number of Rings <7, Size of Biggest Ring <12, 0<tPSA <150, −4<logP <6.0. Finally, undesirable moieties and chemical substructures known to interfere during in vitro bioassays were removes such as, quinone, epoxide, michael acceptors and nitroso.

### Chemicals and antibodies

Small molecules were purchased from ChemBridge Corp. San Diego, USA, and stock solutions were prepared at 10 mM in DMSO, except for compound referred to as **C-13** (methyl 2-{5-[(3-benzyl-4-oxo-2-thioxo-1,3-thiazolidin-5-ylidene) methyl]-2-furyl}benzoate, ChemBridge ID number 6197026) which was prepared at 10 mM stock solution in DMF. All reagents unless otherwise mentioned were from Sigma. The hapten dinitrophenyl (DNP) was purchased from Calbiochem. HRP-conjugated mAb 9E10 was from Santa Cruz Biotechnology. The anti-Syk scFv G4G11 was produced as previously described [34].

### Solubility measurements

4 µl of a 10 mM DMSO stock solution from of each sample were dissolved in 400 µl of PBS and sonicated for 5 minutes. The samples were centrifuged for 15 minutes at 4000 rpm at 25°C, then 250 µl of each sample were transferred to a 96 well plate. 10 µl of each sample were analyzed by HPLC and the sample concentrations were calculated against a series of caffeine standards ranging from 1 to 100 µM.

### Antibody Displacement Assay

Recombinant GST:Syk 6-242 fusion protein [34] was immobilized on an ELISA plate at final concentration of 10 µg ml^−1^. For the screening assay, small molecules diluted in PBS at final concentration of 10 µM were added to the wells for one hour at RT, before adding myc-tagged scFv G4G11 at final concentration of 100 nM for one additional hour. The binding of G4G11 to Syk was evaluated by adding HRP-conjugated mAb 9E10 which detects the amino acid sequence EQKLISEEDLN of human c-myc protein located at the C-terminal end of the scFv.

### Cells, culture conditions and functional assays

The mouse IgE anti-DNP monoclonal antibody 2682-I was used as hybridoma culture supernatants which contained 1 µg ml^−1^ IgE. RBL-2H3 rat basophilic leukaemia cells (ATCC) were maintained as monolayer cultures in RPMI 1640 medium supplemented with 10% fetal bovine serum (Gibco). Measurements of β-hexosaminidase release in RBL-2H3 cells were performed as described [9], except that after 12–16 h incubation with anti-DNP IgE (0.5 µg ml^−1^), cells were incubated for 90 min at 37°C in RPMI medium supplemented with the indicated concentrations of small molecules or DMSO (0.25%). Cells were challenged for 45 min with DNP-BSA (50 ng ml^−1^).

## Supporting Information

Table S1 **The 85 most active compounds selected after** ***in silico*** **and** ***in vitro*** **screen.** For each compound, the ChemBridge ID and the cluster to which it belongs are reported. Direct binding to Syk of some compounds was measured using fluorescence spectroscopy (Kd: dissociation constant). Antibody inhibition percentage reflects the capacity of each compound to inhibit the binding of scFv G4G11 to Syk (final concentrations of 10 µM for small molecules and 100 nM for G4G11). The ability of each compound to inhibit the liberation of allergic mediators from mast cells is measured (**: IC_50_ values of cell degranulation ≥20 µM). Some physicochemical properties computed with FAFDrugs2, as well as the possible ADMET problems detected by our tool are reported. The computations involved: molecular weight (MW), logP, topological polar surface area (tPSA), H-bond acceptors and donors (HBA, HBD).
(PDF)
