# Supplementary material for: Tyrosine Kinase Syk Non-Enzymatic Inhibitors and Potential Anti-Allergic Drug-Like Compounds Discovered by Virtual and In Vitro Screening
Source: PLoS One. 2011 Jun 20;6(6):e21117. doi: 10.1371/journal.pone.0021117 (PMC3118801; doi:10.1371/journal.pone.0021117)
Supplement: Table S1 — The 85 most active compounds selected after in silico and in vitro screen. For each compound, the ChemBridge ID and the cluster to which it belongs are reported. Direct binding to Syk of some compounds was measured using fluorescence spectroscopy (Kd: dissociation constant). Antibody inhibition percentage reflects the capacity of each compound to inhibit the binding of scFv G4G11 to Syk (final concentrations of 10 µM for small molecules and 100 nM for G4G11). The ability of each compound to inhibit the liberation of allergic mediators from mast cells is measured (**: IC50 values of cell degranulation ≥20 µM). Some physicochemical properties computed with FAFDrugs2, as well as the possible ADMET problems detected by our tool are reported. The computations involved: molecular weight (MW), logP, topological polar surface area (tPSA), H-bond acceptors and donors (HBA, HBD). (PDF) [file pone.0021117.s001.pdf]

Table S1.

ID ChemBridge	Cluster	Kd (µM)	Antibody Inhibition	IC50 Cell Degranulation	MW	logP	tPSA	HBD	HBA	State / FAFdrug2	Detected Problems
**6752784**	1	5	86.5 %	**	496.94	5.57	79.73	1	6	Accepted	-
**6670340**	1	16.3	81%	**	453.51	3.02	122.13	1	8	Accepted	-
**6422575**	single	6.2	81%	**	497.31	4.1	94.01	1	7	Rejected	Max Ring Size
**6882059**	1	9.4	79.5 %	**	451.53	3.35	76.27	2	7	Accepted	-
**7111786**	1	-	77.5 %	**	447.48	3.01	93.89	1	8	Accepted	-
**6203863**	1	6.1	73.5 %	**	487.50	4.71	102.37	1	8	Accepted	-
**7347627**	1	-	72%	**	451.53	3.35	76.27	2	7	Accepted	-
**7489416**	1	-	71%	**	514.38	5.27	115.31	2	6	Accepted	-
**6719738**	1	-	71%	**	480.48	5.04	79.73	1	6	Accepted	-
**6650234**	1	6.3	71%	**	480.50	5.33	125.04	1	7	Accepted	-
**6652639**	1	-	70.5 %	**	444.47	3.72	88.96	1	7	Accepted	-
**6673225**	1	-	69%	**	478.51	4.84	134.27	1	8	Accepted	-
**6800873**	single	4.6	67.5 %	**	467.51	4.78	76.07	1	6	Accepted	-
**6879058**	1	-	67.5 %	**	438.49	1.92	89.16	2	8	Accepted	-
**6282824**	2	5.6	67.5 %	5 µM	458.53	4.15	111.41	1	7	Accepted	-
**6750319**	1	-	67%	**	448.49	4.86	125.04	1	7	Accepted	-
**6474819**	single	17.9	63%	**	429.57	5.63	38.53	2	4	Accepted	-
**6498669**	3	0.8	63%	**	467.53	2.78	127.18	3	8	Accepted	-
**6651552**	1	-	60%	**	467.55	4.24	67.04	2	6	Accepted	-
**6453860**	3	-	60%	**	454.51	5.65	76.66	2	6	Accepted	-
**6853966**	3	-	58%	**	469.51	2.4	131.21	3	9	Accepted	-
**6866968**	single	-	57.5%	**	464.61	3.82	61.78	3	6	Accepted	-
**7938324**	single	-	57%	**	444.52	3.49	107.5	0	6	Accepted	-
**6905988**	1	-	57%	**	455.49	3.08	76.27	2	7	Accepted	-
**6885782**	1	-	56.5%	**	532.00	3.55	94.73	2	9	Accepted	-
**6663684**	1	-	56.5%	**	435.53	3.78	67.04	2	6	Accepted	-
**6672500**	1	-	55%	**	487.95	3.22	85.5	2	8	Accepted	-
**7721949**	1	-	54%	**	453.55	4.07	76.27	2	7	Accepted	-
**7966545**	2	6.7	53.5%	**	486.52	3.33	123.27	2	8	Accepted	-
**7437580**	single	-	51%	**	481.56	3.0	125.46	2	8	Accepted	-
**6654239**	1	-	50%	**	471.49	2.7	85.5	2	8	Accepted	-
**6670570**	1	-	50%	**	500.58	1.8	112.8	2	9	Accepted	-
**6670673**	1	-	50%	**	471.49	2.7	85.5	2	8	Accepted	-
**6670747**	1	-	50%	**	437.50	2.99	76.27	2	7	Accepted	-
**6671401**	1	-	50%	**	443.46	1.99	98.64	2	9	Accepted	-
**6677533**	1	-	50%	**	459.53	2.61	113.74	2	8	Accepted	-
**6683618**	1	-	50%	**	457.92	3.25	76.27	2	7	Accepted	-
**6417902**	single	-	49.5%	**	478.62	4.19	66.27	1	6	Accepted	-
**6437157**	single	-	49.5%	**	461.89	5.48	80.75	0	6	Accepted	-
**6465972**	3	-	49%	**	484.56	4.17	102.55	1	8	Accepted	-
**7723671**	1	-	48.5%	**	485.52	3.05	85.5	2	8	Accepted	-
**6648368**	1	-	48.5%	**	459.91	3.74	67.04	2	6	Accepted	-
**6656195**	1	-	48.5%	**	453.52	3.88	67.04	2	6	Accepted	-
**7778331**	single	3	48%	**	475.58	1.73	105.55	3	9	Accepted	-
**6994060**	2	-	47.5%	**	485.98	3.15	115.85	2	8	Accepted	-
**6458830**	3	-	47%	**	492.65	4.56	66.27	1	6	Accepted	-

**7524107**	2	5.5	46.5%	**	445.48	5.2	104.4	2	7	Accepted	-
**6661524**	1	-	46.5%	**	541.46	3.79	103.57	2	8	Accepted	-
**7739436**	1	-	46%	**	438.49	2.86	89.16	2	8	Accepted	-
**6881804**	1	-	46%	**	445.48	2.74	98.64	2	9	Accepted	-
**6907921**	1	-	45.5%	**	427.47	2.42	89.41	2	8	Accepted	-
**7495334**	2	1.6	43%	10 µM	450.46	2.69	145.65	1	9	Accepted	-
**7509862**	4	-	42.5	10 µM	515.58	4.37	131.3	2	8	Intermediate	Flagged Group Nitrile
**7325385**	1	-	42%	**	495.58	3.68	85.5	2	8	Accepted	-
**6669449**	4	-	42%	**	424.40	4.39	127.16	3	8	Intermediate	Flagged Group Nitrile
**7348779**	single	-	40%	**	438.55	4.58	59.08	0	6	Accepted	-
**7501888**	2	8.2	40%	4 µM	539.04	5.27	112.8	1	8	Accepted	-
**6946138**	4	-	40%	**	415.41	5.47	85.64	0	6	Accepted	-
**7722851**	5	21.8	39.5%	4 µM	523.00	3.59	128.09	1	8	Accepted	-
**7517583**	single	-	38.5%	**	476.47	2.22	102.45	0	9	Accepted	-
**6634701**	single	-	38.5%	10 µM	470.54	4.23	102.74	0	7	Rejected	-
**7726450**	1	-	37.5%	**	458.46	2.74	110.69	1	9	Accepted	-
**6662088**	1	-	37%	**	515.60	1.09	125.69	2	10	Accepted	-
**7752193**	2	8	35%	10 µM	451.54	3.1	115.85	2	8	Intermediate	Flagged Group Alkene
**7238569**	single	-	32%	**	478.56	3.54	132.82	4	8	Accepted	-
**7724000**	1	-	30%	**	469.48	2.92	85.5	2	8	Accepted	-
**7443270**	2	-	30%	10 µM	451.49	3.26	120.89	1	8	Accepted	-
**7245019**	single	-	30%	**	428.47	4.99	76.05	1	6	Accepted	-
**6909597**	1	-	29%	**	457.92	3.25	76.27	2	7	Accepted	-
**7661882**	2	-	28%	**	443.49	4.01	74.86	1	7	Accepted	-
**7667791**	single	-	28%	**	434.48	3.5	82.21	2	7	Accepted	-
**6891745**	1	-	27.5%	**	467.53	2.95	85.5	2	8	Accepted	-
**7783660**	2	4.8	27%	10 µM	507.60	3.74	132.92	2	9	Intermediate	Flagged Group Alkene
**7653478**	single	-	27%	**	451.54	3.12	91.36	2	9	Accepted	-
**7723330**	5	-	26%	**	501.48	2.88	114.48	1	10	Accepted	-
**7199725**	2	-	25%	**	440.55	4.62	49.61	2	5	Accepted	-
**6987235**	single	-	25%	**	476.00	5.28	50.77	0	4	Rejected	Max Ring Size
**7140931**	single	-	24.5%	**	472.59	3.47	92.37	0	7	Accepted	-
**7787455**	3	-	20%	**	454.49	3.36	110.39	1	8	Accepted	-
**7660465**	single	3.2	20%	15 µM	536.87	4.33	106.62	2	7	Accepted	-
**7661751**	1	-	19%	**	466.50	1.66	98.39	2	9	Accepted	-
**7722914**	5	15.6	19%	15 µM	513.62	2.29	116.79	2	9	Accepted	-
**7745040**	1	-	18%	**	542.39	4.05	89.41	2	8	Accepted	-
**7735385**	1	-	17%	**	464.53	3.79	93.07	2	8	Accepted	-
**7756003**	single	-	11%	10 µM	484.61	4.5	104.57	0	7	Accepted	-
